# Predictors of Metabolic Syndrome in Polish Women—The Role of Body Composition and Sociodemographic Factors

**DOI:** 10.3390/jcm14165911

**Published:** 2025-08-21

**Authors:** Katarzyna Dereń, Magdalena Zielińska, Anna Bartosiewicz, Edyta Łuszczki

**Affiliations:** Faculty of Health Sciences and Psychology, Collegium Medicum, University of Rzeszów, 35-959 Rzeszów, Poland

**Keywords:** body composition, obesity, obesity prevention, metabolic syndrome, women

## Abstract

**Background:** The increasing prevalence of overweight and obesity worldwide is one of the most serious public health challenges, reaching epidemic proportions. Excessive body weight is often accompanied by metabolic disorders such as insulin resistance, atherogenic dyslipidaemia and hypertension—collectively known as metabolic syndrome. This cross-sectional study aimed to identify predictors of metabolic syndrome in women using logistic regression analysis based on selected sociodemographic, anthropometric, and lifestyle variables. **Methods**: The study included 250 women aged 23–85 recruited in the Podkarpackie region of Poland. Data on sociodemographic characteristics and smoking status were collected via a questionnaire. Physical activity and sedentary behaviours were assessed using the Global Physical Activity Questionnaire. Body composition was measured using bioelectrical impedance analysis. Fasting capillary blood samples and blood pressure measurements were obtained by qualified medical staff in accordance with standard procedures. **Results**: Obesity was strongly associated with metabolic syndrome components, particularly abnormal blood pressure (66.3%) and fasting glucose (64%), both of which were statistically significant (*p* < 0.01). Age was a significant predictor of metabolic syndrome (OR = 1.06; *p* < 0.01) and its components, including hypertension, dysglycaemia and dyslipidaemia. Waist-to-hip ratio was strongly linked to metabolic syndrome (OR = 356.97; *p* < 0.01) and obesity (OR = 5.89 × 10^30^; *p* < 0.001); however, these exceptionally high values should be interpreted with caution, as they may reflect statistical artifacts due to model instability or sample characteristics, rather than a meaningful or generalizable association. Higher body fat mass was associated with an increased risk of obesity, hypertension and dysglycaemia (OR = 1.42, 1.06 and 1.06 respectively; *p* < 0.01). **Conclusions:** These results emphasise the significant role of obesity as a risk factor for metabolic syndrome in women, highlighting the need for personalised preventive strategies that consider lifestyle and sociodemographic factors, such as targeted health education, promotion of physical activity, and dietary counselling adapted to the needs of women at risk.

## 1. Introduction

The increasing prevalence of overweight and obesity worldwide is one of the most serious public health challenges, reaching epidemic proportions [1,2]. Excessive body weight is often accompanied by metabolic disorders such as insulin resistance, atherogenic dyslipidaemia and hypertension [3]. The occurrence of these conditions together is referred to as metabolic syndrome (MetS), which significantly increases the risk of developing type 2 diabetes and cardiovascular disease (CVD). This issue is of particular importance given its steadily increasing prevalence [4,5].

It is estimated that MetS affects between 12.5% and 31.4% of adults worldwide, depending on the region and the diagnostic criteria used [6]. Its prevalence increases with age and differs by sex being more common in men under 50 and in women over 50 [7]. In Poland, a representative population-based study reported a MetS prevalence of 31.7%, based on the updated 2022 national diagnostic criteria [8]. These diagnostic criteria include obesity, which is defined as a body mass index (BMI) of at least 30 kg/m^2^ or a waist circumference of at least 88 cm for women and at least 102 cm for men, as well as at least two of the following three factors: abnormal glucose metabolism, atherogenic dyslipidaemia (elevated non–high-density lipoprotein cholesterol (non-HDL)), or elevated blood pressure. The pharmacological treatment of any of these conditions is also considered during the diagnostic process [9]. Notably, the adoption of these new criteria resulted in a decreased prevalence of MetS among Polish men, with minimal change observed in women [8].

Importantly, obesity, particularly central obesity, is a key risk factor for MetS [8,9]. Poland has also experienced a consistent rise in excess weight, with predictions suggesting that obesity could affect up to 33% of Polish adults by 2035 [10]. The likelihood of obesity is twice as high for women than for men [11]. This phenomenon has a multifactorial aetiopathogenesis that includes sociocultural factors, such as dietary patterns and social expectations; economic factors, such as income level, educational attainment, and living environment; and biological factors, including genetic predisposition, metabolic changes associated with pregnancy, and variations in hormonal balance [12,13,14,15,16]. A meta-analysis by Censin et al. showed that BMI has a significantly greater impact on the risk of developing type 2 diabetes in women than in men [15]. Furthermore, MetS is associated with a higher relative risk of CVD in women than in men. Large cohort studies have shown that the risk ratios for CVD mortality associated with MetS are significantly higher in women [17]. Therefore, targeted analyses of MetS in the female population are necessary, as identifying its predictive factors could be key to developing preventive strategies for MetS and obesity.

Although international studies primarily focus on the anthropometric, sociodemographic, biological, reproductive and psychosocial factors associated with MetS, there is a lack of in-depth research in Poland aimed at identifying its key determinants [18,19,20,21,22]. Therefore, the aim of this cross-sectional study was to identify predictors of MetS in women using logistic regression analysis, which considered selected sociodemographic, anthropometric and lifestyle variables. We hypothesized that (1) the prevalence of MetS increases significantly with increasing BMI, and is highest among women with obesity, and (2) age, education level, place of residence, occupational activity, physical activity (PA), sedentary behaviours (SB), smoking status, body fat mass (BFM), and the waist-hip ratio (WHR) are significant predictors of MetS and its components. The findings of this study may contribute to the identification of high-risk groups and support the development of more effective, integrated prevention strategies that address the multifactorial nature of MetS and obesity, along with their associated health consequences.

## 2. Materials and Methods

### 2.1. Study Group

A cross-sectional study recruited 250 women aged between 23 and 85 who were enrolled at various study sites located in the Podkarpackie province of Poland. Participants were selected using a combination of purposive and convenience sampling. Interested participants received detailed study information and were screened for eligibility through a brief questionnaire and interview conducted by trained research staff. The inclusion criteria were as follows: female sex; age between 18 and 85 years; not being currently pregnant or breastfeeding, due to hormonal and metabolic changes that could affect study variables; no diagnosed mental or neurological disorders (including schizophrenia, epilepsy, or dementia); no use of medications known to significantly affect metabolic parameters, such as systemic corticosteroids, antipsychotics, or anti-obesity agents; and provision of written informed consent. Exclusion criteria included: age below 18 or above 85 years; current pregnancy or lactation; use of pharmacological agents known to interfere with metabolic processes; and any contraindications to bioelectrical impedance analysis, such as the presence of a pacemaker, metal implants, or severe fluid retention. The study was conducted in 2023–2024 as part of a project in which our team also conducted research focusing on the relationship between overweight/obesity and factors such as food addiction, SB, and mental health. The project also involved analysing the prevalence of MetS, as well as differences in anthropometry and behaviour according to age [23,24]. The aforementioned previous publications by our team provide a detailed description of the methodology used.

### 2.2. Sociodemographic and Lifestyle Data

The following general information was gathered using a questionnaire: gender; age; level of education; place of residence; professional activity; use of pharmacotherapy; and smoking habits [25]. Levels of PA and SB were assessed using the Global Physical Activity Questionnaire (GPAQ), developed by the World Health Organization (WHO) [26,27]. This tool integrates elements of both the short and long forms of the International Physical Activity Questionnaire (IPAQ) and comprises 16 items covering four key domains: activity performed at work, active transportation (e.g., walking or cycling for at least 10 min continuously), recreational PA, and sedentary time during a typical day. The collected data were processed according to WHO protocol enabling estimation of total weekly PA expressed in metabolic equivalent minutes (MET-min/week) [28]. Participants were categorized as insufficiently active if they did not meet WHO-recommended threshold i.e., at least 150 min of moderate-intensity activity, 75 min of vigorous-intensity activity, or a combination thereof, yielding a minimum of 600 MET-min/week. Based on these criteria, respondents were classified into low, moderate, or high PA levels. The GPAQ is a standardized and widely used instrument with demonstrated reliability and validity across diverse populations and settings.

### 2.3. Anthropometric Measurements

Body composition analysis was performed using electrical bioimpedance with a body composition analyser (Tanita BC-418 MA, Tokyo, Japan). The measurement was taken in accordance with the applicable standard, taking into account the exclusion criteria for this method [29]. To minimize the influence of hydration status, all measurements were performed in the morning after an overnight fast. Participants were instructed to abstain from alcohol and vigorous PA for at least 24 h prior to the assessment and to avoid consuming large amounts of fluids on the morning of the measurement. Waist and hip circumferences were measured twice to the nearest 0.1 cm using a SECA tape measure. The average of the two measurements was taken for further analysis. Based on the obtained data, WHR was calculated using the following formula: WHR = waist circumference (cm)/hip circumference (cm) [30].

### 2.4. Biochemical Measurements and Blood Pressure Measurements

Fingertip capillary blood samples were collected from study participants in the morning, while they were fasting, by qualified medical personnel, in accordance with current procedures. Results for total cholesterol (TC), high-density lipoprotein (HDL), low-density lipoprotein (LDL) and triglycerides (TG) were obtained using a Cardiocheck PA analyser. Non-HDL cholesterol was calculated using the formula: non-HDL = TC − HDL. Fasting blood glucose levels were measured using a Contour TM Plus One glucose meter. Blood pressure was measured three times using a Welch Allyn 4200B-E2 automatic sphygmomanometer (Aston Abbotts, UK) according to European Society of Hypertension (ESH) guidelines [31]. The average of these three measurements was taken for analysis.

### 2.5. Criteria for Diagnosis of MetS

The latest Polish MetS diagnostic criteria, which were developed by experts from Polish scientific societies and published in 2022, were used in the present study. A MetS diagnosis was made if either obesity (BMI ≥ 30 kg/m^2^) or abdominal obesity (waist circumference ≥ 88 cm in women) was present, and if two of the following three criteria were met: (1) a fasting plasma glucose level of at least 100 mg/dL, or the use of hypoglycaemic treatment; (2) a non-HDL cholesterol level of at least 130 mg/dL, or the use of hypolipidaemic treatment; and (3) a blood pressure level of at least 130/85 mmHg, or the use of hypotensive treatment [9]. These criteria were operationalized using measured values of BMI, waist circumference, fasting plasma glucose, non-HDL cholesterol, and blood pressure, as well as participants’ self-reported use of hypoglycaemic, hypolipidaemic, or hypotensive medications.

### 2.6. Statistical Analysis

The significance level was set at *p* = 0.05. Variables expressed at the ordinal or nominal level were analysed using chi-squared tests based on distribution. Continuity correction was applied to 2 × 2 tables. If the conditions for the chi-squared test were not met, Fisher’s exact test with expansion was used for tables larger than 2 × 2. Logistic regression analysis was used to verify the existence of a relationship between the variables. The calculations were performed using the R statistical environment (version 3.6.0), the PSPP program, and MS Office 2019. Prior to analysis, the dataset was checked for completeness. Participants with missing or incomplete data in key variables were excluded, and all analyses were conducted on complete cases only.

An a priori power analysis was conducted using G*Power software (version 3.1.9.7) to estimate the minimum sample size required to detect statistically significant effects in logistic regression. The parameters were set as follows: two-tailed test, *α* = 0.05, power = 0.80, medium effect size (odds ratio = 1.5), base event probability = 0.36 (based on the observed MetS prevalence in the sample), and R^2^ of other predictors = 0.10. Under these assumptions, the required sample size was estimated at 248 participants. As the actual sample included 250 participants, the study had sufficient power to detect effects of at least medium magnitude.

### 2.7. Ethics Approval and Consent to Participate

The present study was conducted in accordance with the guidelines for cross-sectional studies and in accordance with the Declaration of Helsinki and was approved by the Bioethics Committee of the University of Rzeszów (Resolution No. 2023/07/0046 of 25 June 2023). All participants were informed about the purpose of the study and assured of their anonymity. Participation was preceded by the obtaining of written informed consent.

## 3. Results

Most of the women lived in urban areas (81.2%), were employed (69.6%) and had a higher education (68.4%). Obesity to varying degrees was present in 34.4% of the participants and overweight in 32.4%. An insufficient level of PA was declared by 44.4% of the participants and smoking by 17.2%. Regarding biochemical and blood pressure parameters, abnormal results predominated for TC (65.6%) and non-HDL (61.6%). Criteria for MetS were met by 36.4% of women. Table 1 shows the sociodemographic and health characteristics of the study group (N = 250).

The Table 2 shows the basic descriptive statistics (minimum, maximum, median) for the anthropometric and biochemical variables in the group of female subjects (N = 250). The mean age of the female subjects was 54.79 years (*SD* = 13.49), and the mean BMI was 28 kg/m^2^ (*SD* = 5.92).

Due to the small numbers of each category of BMI ranges, the category of grade I obesity was merged with the categories of grade II obesity and grade III obesity. The underweight category was excluded completely. The results of the analysis shown in Table 3 indicate that obesity was strongly associated with the presence of individual MetS criteria. Women with obesity were most likely to meet the criteria of abnormal blood pressure (*χ*^2^ (2, N = 250) = 19.27; *p* < 0.01) and abnormal fasting glucose levels (*χ*^2^ (2, N = 250) = 21.56; *p* < 0.01), among others, with percentages of 66.3% and 64%, respectively, in contrast to the normal-weight and overweight groups (*p* = 0.001). The only criterion that showed no significant differences between the groups was the non-HDL level (*χ*^2^ (2, N = 250) = 0.11; *p* > 0.05).

The logistic regression model was statistically significant, indicating that the selected predictors effectively explain the variation in MetS incidence (R^2^ = 0.32, *χ*^2^ (9, N = 222) = 94.26, *p* < 0.001). BMI was excluded from the model due to excessive collinearity. The remaining variables were characterised by variance inflation factors (VIFs) of less than 2.5 and a tolerance of greater than 0.4, which indicates the absence of significant collinearity and the stability of the regression models. Detailed values are provided in the Supplementary Material (Table S1).

Age was a significant predictor of MetS (OR = 1.06; *p* < 0.01) and each of its components, i.e., hypertension (OR = 1.06; *p* < 0.001), dysglycaemia (OR = 1.05; *p* < 0.001) and dyslipidemia (OR = 1.04; *p* < 0.01). WHR was strongly associated with the risk of MetS (OR = 356.97; *p* < 0.01) and obesity (OR = 5.89 × 10^30^; *p* < 0.001), while BFM significantly increased the likelihood of obesity (OR = 1.42; *p* < 0.001), abnormal blood pressure and dysglycaemia (both OR = 1.06; *p* < 0.01). WHR was the strongest predictor of MetS risk. An increase in WHR was associated with an almost 357-fold increase in the odds of MetS. However, such an extreme odds ratio may indicate quasi-complete separation or structural instability in the model, likely due to a small number of participants with low WHR among those with MetS.

Residing in an urban area was associated with a higher risk of MetS (OR = 2.53; *p* < 0.05) and abnormal blood pressure (OR = 2.65; *p* < 0.05). Furthermore, employment status was significantly associated with abnormal non-HDL cholesterol levels (OR = 5.90; *p* < 0.001). Higher education was associated with a lower risk of abnormal blood pressure (OR = 0.40; *p* < 0.05). Total PA, cigarette smoking, and SB showed no significant association with the incidence of MetS or its individual components, except for a slight effect of SB on dyslipidemia (*p* < 0.05). Table 4 shows the odds ratios (OR) with levels of statistical significance for selected sociodemographic variables, lifestyle and anthropometric parameters.

Diagnostic criteria based on Polish 2022 guidelines. Each component includes either the measured parameter (above threshold) or the corresponding pharmacotherapy.

Table 5 shows the predicted probabilities of MetS in women according to values of age, BFM and WHR. The lowest probability was recorded for women aged 42.55 years, with BFM = 18.50 kg and WHR = 0.73 (95% CI: 0.00–0.07; SE = 0.01; *p* = 0.02), and the highest for women aged 68.93 years, with BFM = 37.18 kg and WHR = 0.97 (CI: 0.67–0.90; SE = 0.06; *p* = 0.81). Thus, the older the respondents were, and the higher their BFM and WHR, the significantly higher the likelihood of MetS. Additional model-predicted probabilities for MetS and its components across key predictor combinations are provided in Supplementary Material (Tables S2–S6).

## 4. Discussion

The international literature often emphasizes that advanced age, lower levels of education, residence in urban areas, limited PA and higher BMI are important risk factors for the development of MetS [32,33,34].

The results obtained in this study are partially consistent with observations reported in the international literature. In our study, age was one of the strongest predictors of MetS and its components. Each additional year of life was accompanied by a 6% increase in MetS risk, abnormal blood pressure by 4%, abnormal fasting glucose by 5% and abnormal lipid profile by 4%. Similar relationships have also been reported in studies conducted in Poland. Szostak-Węgierek et al. in a study of non-pregnant Polish women of childbearing age (20–49 years), observed an increasing prevalence of overweight, obesity—especially abdominal obesity—as well as MetS and other metabolic disorders with age [35]. Łuszczki et al. demonstrated in a study of Polish women that the prevalence of MetS was higher in women with obesity aged 65 and older compared to younger women [24]. According to the WOBASZ II study in Poland, the prevalence of MetS in Poland peaks in the 60–79 age group, which is consistent with our results showing that in the study group of women, the highest risk of MetS among women was at age 69, with higher WHR and BFM [36]. The risk of developing metabolic disorders increases with age and may be due to chronic inflammation, hormonal changes, and deteriorating body composition [37]. Given the increasing life expectancy and projected growth of the elderly population (expected to reach 1.6 billion by 2050), it is crucial to address the issue of age-related metabolic disorders [38,39].

The rising incidence of MetS highlights the importance of analysing sociodemographic determinants. A meta-analysis by Blanquet et al. found that a low socioeconomic status (including lower education, lower income, and unemployment, among other factors) significantly increases the risk of MetS [40]. Yi and An identified socioeconomic status, including education level and household income, as a major risk factor for MetS in women [41]. The data obtained in this study are consistent with these observations. Higher education was shown to have a protective effect on components of the MetS, reducing the risk of abnormal blood pressure by about 60%. The literature emphasizes that people with higher education are more likely to engage in health-promoting behaviors, such as regular PA, reduced sodium intake, and better weight and stress control, and all of these factors significantly affect the blood pressure profile [42,43,44,45]. Higher education may also be associated with better health awareness of hypertension [46]. In addition to education level, place of residence may also be an important factor in differentiating metabolic risk.

This study found that women living in urban areas were more than twice as likely to develop MetS and abnormal blood pressure as rural residents. However, Kim and Chao obtained different results in a study conducted among women over 50, indicating that rural women were more likely to develop MetS than their urban peers [47]. Similar observations were made by Nowicki et al., who conducted a cross-sectional study of 4040 people living in eastern Poland and found a higher prevalence of MetS among rural residents. Regardless of gender, MetS was diagnosed more frequently in rural residents than in urban residents [48]. A cross-sectional study conducted by Jiao et al. among individuals aged ≥50 years in northwest China demonstrated that rural women also had a significantly higher prevalence of MetS (53.6%) compared to their suburban (52.0%) and urban (45.5%) counterparts (*p* = 0.003) [49]. The authors identified rural residence, age, weight gain, and limited dietary control as independent risk factors. Interestingly, rural women exhibited higher central obesity and hypertension despite having a lower family history of metabolic diseases, which could reflect lifestyle changes driven by economic development. A meta-analysis by Nsabimana et al., involving over 313,000 participants from low- and middle-income countries, confirms a higher risk of MetS among urban residents compared to rural residents [50]. According to the results of the meta-analysis, the observed differences may be significantly influenced by confounding factors such as educational level, socioeconomic status, lifestyle, access to processed foods and PA. Another systematic review and meta-analysis found that the mean prevalence of MetS was 22.5% in urban populations, compared to 12.0% in rural areas, indicating a markedly higher risk of MetS among urban residents [51]. Similar results were obtained in a recent study conducted in Rwanda by Gafirita et al., in which the prevalence of MetS was significantly higher among urban residents (57.1%) than rural residents (42.9%) [52]. The observed discrepancies may reflect regional variability in urbanization, socioeconomic development, and cultural or dietary patterns. In addition to broader demographic and environmental differences, individual-level factors such as women’s occupational status, income, psychosocial stress, healthcare access, and unmeasured lifestyle habits (e.g., diet, PA) may have contributed to the observed associations. These potential confounders highlight the need for further regionally stratified and well-controlled studies.

In the present study, working women showed an almost six-fold higher likelihood of having elevated non-HDL cholesterol. While this association is statistically significant, it should be interpreted with caution, as it may be confounded by unmeasured factors such as work-related stress, occupational type, diet, or lifestyle habits. The cross-sectional nature of the study further limits any causal interpretation. A similar relationship was observed by Kim and Chao, who showed a higher risk of MetS in active women [47]. A similar context was investigated in a study by Raczkiewicz et al. conducted among 300 Polish women aged 44–66 years with intellectual jobs, in which the prevalence of MetS was 23.7%. The authors indicated that the risk of MetS correlated primarily with BMI, amount of body fat and number of pregnancies, while factors such as age, education, place of residence, marital status or use of hormone replacement therapy had no significant effect on the incidence of MetS [53]. Our observations may be explained by the mechanisms described by the European Atherosclerosis Society [54]. During the perimenopausal period, women experience adverse metabolic changes, including increased concentrations of TC, LDL and non-HDL, which increases cardiovascular risk. The authors also emphasise the role of psychosocial risk factors, including chronic stress, which tends to affect women with multiple social roles, particularly professional ones. Given the evidence suggesting that non-HDL is a significant predictor of cardiovascular events in women, particularly under the age of 55, these findings warrant further investigation in studies involving a larger sample size. The latest review by Katsi et al. has extensively discussed the significance of elevated non-HDL cholesterol as an indicator of increased cardiovascular risk [55]. Non-HDL-C includes the cholesterol found in all atherogenic lipoproteins (including LDL, very-low-density lipoprotein (VLDL), intermediate-density lipoprotein (IDL) and lipoprotein (a)) and provides a more accurate reflection of total atherosclerotic risk than LDL does, particularly in individuals with mixed dyslipidaemia, hypertriglyceridaemia, obesity or insulin resistance. Notably, women, particularly postmenopausal women, exhibit an increase in non-HDL and apolipoprotein B (Apo B), even when LDL values are normal. This can result in an underestimation of risk when relying on traditional markers. In light of these data, our results may reflect the biologically justified phenomenon of increased atherogenic particle burden in working women. A study by Chaudhuri and Maulik, conducted among young female teachers, identified a link between occupational stress and an unfavourable lipid profile. High stress levels were found to correlate with increased concentrations of TC, LDL and TG. A three-month relaxation intervention involving progressive muscle relaxation (PMR) was found to improve all lipid parameters [56]. These results suggest a potential psychoneuroendocrine mechanism whereby chronic occupational stress in women could contribute to dyslipidaemia. A large cohort study involving 5000 manual workers also confirmed the relationship between occupational stress and lipid disorders, showing that even mild psychological stress significantly increases the risk of an abnormal lipid profile [57]. This effect was independent of BMI and age, suggesting the existence of an additional stress-related mechanism affecting lipid profiles. These results are consistent with our observation of poorer lipid profiles in professionally active women who experience chronic stress related to work and their various social roles. Our findings may be also related to lifestyle characteristics of working women, including higher sedentary work patterns, low levels of PA, or less favorable eating habits [58,59,60,61].

Although sociodemographic factors can modify metabolic risk, anthropometric parameters are key predictors of MetS, providing information about the body’s metabolic status. In our study, both BFM and WHR were found to be significant predictors of MetS and its components. An increase of 1 kg in BFM was associated with a 15% higher risk of MetS, a 42% higher risk of obesity, and a greater likelihood of hypertension and hyperglycaemia. WHR, however, was the strongest predictor in the model, emphasising the significance of visceral obesity in the development of MetS.

Several studies have shown that BMI is a key predictor of MetS [62,63]. Notably, the study by Van Ancum et al., despite analysing a wide range of variables (clinical, lifestyle and physical fitness), did not identify any predictors that were stronger than BMI [62]. However, our findings suggest that BMI alone may not fully capture the risk of MetS. In our study, some women with a normal BMI still met the diagnostic criteria for MetS, highlighting the limitations of BMI as a standalone indicator. This observation aligns with previous research showing that MetS can occur in individuals with a normal body weight [64]. Although BMI is a useful and widely used indicator, it is worth remembering that it does not reflect qualitative differences in body fat distribution. Our results confirm this: WHR and BFM were found to be the strongest predictors of MetS risk. It should be noted that the odds ratio for WHR was extremely high (OR = 356.97), which may reflect overfitting or near-perfect separation in the logistic model. While WHR was a significant predictor of MetS, this result should be interpreted with caution, particularly given the relatively small sample size and potential outliers. Future research should explore this relationship using larger samples and statistical methods robust to separation issues.

A study by Bartosiewicz et al., conducted among nurses in Poland, showed that overweight or obesity were the strongest predictors of MetS—the risk increased by a factor of over eight in the case of being overweight, and by a factor of over sixteen in the case of grade II/III obesity, compared to women of normal weight [65]. Similarly, a multicentre study by Babicki involving 672 Polish women aged 35 years or over, with no history of cardiovascular disease or diabetes, showed a significant association between abdominal obesity and the prevalence of MetS [66]. The relationship between body composition and MetS has also been confirmed in international studies. The study by Pouragha et al. demonstrated that fat mass, visceral fat, muscle mass and lean mass are all strongly associated with an increased risk of MetS, indicating the diagnostic value of anthropometric parameters [67]. Gutierrez-Esparza et al., in turn, identified key predictors of MetS in women, such as waist circumference [68]. A meta-analysis by Krishnamoorthy et al., which covered the adult population of India, also highlighted a strong association between anthropometric indicators, such as overweight or obesity, and the occurrence of MetS. This confirms the important role of body parameters as markers of metabolic risk in different populations [69]. In summary, the results obtained confirm the crucial role of anthropometric parameters, particularly visceral obesity, in predicting MetS. This emphasises the need to identify women at risk early on, even in a population generally considered healthy, by taking into account not only body weight, but also fat distribution. In addition to somatic factors, lifestyle factors such as PA levels and time spent sitting are important when assessing metabolic risk.

A review published in 2024 collected data from 19 studies and found that individuals with the healthiest lifestyles (characterised by regular PA, a balanced diet, and abstinence from smoking) were 43% less likely to develop MetS than those with the least healthy lifestyles [70]. This association was observed in both cross-sectional and cohort studies, in which a healthy lifestyle was consistently linked to a lower prevalence and incidence of MetS. A study based on data from almost 7000 Korean adults revealed a significant correlation between the number of adverse lifestyle factors and the likelihood of developing MetS. The greater the number of unhealthy behaviours a person exhibited (including a SB, poor sleep, alcohol consumption, smoking, and an unhealthy diet), the greater their risk of developing MetS [71]. However, this study found that SB had a small but significant effect on the lipid profile only. Each additional minute spent sitting per day increased the risk of abnormal non-HDL by around 0.2% (*p* < 0.05). No statistical significance was found for total PA. Importantly, longitudinal studies show that higher levels of PA are associated with a lower risk of MetS. For instance, taking part in sports for over 75 min each week has been linked to a 29% reduced likelihood of developing MetS over 12.5 years of follow-up [72]. PA undoubtedly plays a key role in preventing and controlling MetS because it has a beneficial effect on its main components, such as insulin sensitivity, lipid profile, blood pressure, and body composition. The lack of a significant association with total PA in this study may be due to limitations in the way the data was measured (self-reported data), low variability in activity levels among the participants, or the fact that physically active individuals also spend long periods of time sitting down, which cancels out the beneficial effects of exercise.

In this study, smoking was not found to be significantly associated with an increased risk of MetS or its components. This finding is consistent with that of Jamali et al., who also failed to observe such an association when adjusting for confounding variables [73]. Although smoking is widely recognised as an independent risk factor for MetS, particularly in cases of abdominal obesity, dyslipidaemia and insulin resistance, the results of studies in this area are inconsistent [74]. Interestingly, a meta-analysis by Kim and Cho found that quitting smoking could paradoxically increase the risk of developing MetS, particularly among women. This may be due to weight gain and variations in hormonal and lipid metabolism [75]. In contrast, a recent Korean cohort study found that the use of heated tobacco products (HTP) is associated with a significantly higher risk of developing MetS than the use of traditional cigarettes, especially among long-term and heavy users of HTP [76]. These findings challenge the perception of HTP as a healthier alternative and point to the need to include these products in metabolic prevention strategies. It is also worth noting that, in women, smoking is associated with poorer MetS rates and, when combined with other lifestyle factors such as low PA, it increases the risk of MetS, including abdominal obesity and high triglyceride levels [77,78]. Further studies, preferably prospective, are required to better understand the role of smoking in the pathogenesis of MetS, particularly in the female population.

Beyond traditional behavioural and demographic risk factors, growing attention has been directed toward molecular, lipidomic, and metabolomic markers that may underlie the development and progression of MetS and its complications. In particular, sphingolipid metabolites—such as ceramides—have emerged as important mediators linking metabolic dysregulation with vascular dysfunction, inflammation and atherosclerosis development. Their accumulation has been associated with adverse cardiovascular outcomes and may serve as early biomarkers of cardiometabolic risk [79]. This expanding field opens up new avenues for risk stratification and targeted interventions, especially in women, whose cardiometabolic profiles may be influenced by complex lipid–hormonal interactions. In addition, a recent study by Li et al. demonstrated that individuals presenting with a phenotype characterised by an increased WHR and BMI, but with normal metabolic indices, exhibited alterations in their gut microbiota and reduced levels of phytosphingosine. These alterations were associated with an increased risk of future metabolic impairment in experimental models [80]. Complementing these findings, Huang et al. conducted a Mendelian randomization analysis indicating a suggestive negative causal relationship between glycolithocholate (GLCA)—a conjugated bile acid—and both general obesity and trunk fat percentage. Although the associations did not remain statistically significant after multiple comparison correction, the study supports the emerging view that specific bile acid metabolites may play a role in modulating adiposity and metabolic outcomes [81]. Moreover, recent work has highlighted the potential of integrated indices such as the triglyceride-glucose (TyG) index and its obesity-related derivatives (TyG-BMI, TyG-WC, TyG-WHtR) as reliable surrogate markers of insulin resistance and predictors of cardiometabolic risk. In a large-scale NHANES-based analysis, Li et al. demonstrated that these indices are independently associated with both all-cause and cardiovascular mortality in hypertensive individuals. Among them, TyG-WHtR showed the strongest association with mortality, suggesting its utility in risk stratification. Importantly, machine learning approaches confirmed the incremental predictive value of TyG-based indices beyond traditional risk factors, underscoring their potential role in precision medicine [82].

In summary, the results of the logistic regression analysis revealed that age, place of residence, and certain anthropometric parameters were the strongest predictors of MetS risk. This study contributes to the existing body of literature by providing an updated analysis of MetS predictors in a female population, using recently adopted national diagnostic criteria. Unlike many earlier studies that rely primarily on BMI, our findings underscore the predictive strength of WHR and BFM—measures that better reflect fat distribution and body composition. Furthermore, the focus on a Polish female population fills a notable gap in regional data, particularly in the context of sociodemographic and occupational influences on metabolic risk. These insights may inform more precise, gender-sensitive approaches to MetS prevention.

### 4.1. Limitations

The limitations of the present study include its cross-sectional design, which prevents the establishment of cause-and-effect relationships, and the omission of certain potential confounding factors, including sleep quality, alcohol consumption, stress levels, and dietary habits. Several key variables (e.g., PA and smoking status) were self-reported. While validated instruments such as the GPAQ were used, self-reports may be subject to recall bias and social desirability bias. No objective validation methods (e.g., accelerometry for PA or biochemical markers such as cotinine for smoking) were employed. However, smoking status was assessed using a standard self-report approach that is widely accepted in epidemiological studies. While no biochemical validation was performed, previous research has demonstrated good concordance between self-reported smoking status and biomarker-based verification, particularly when participants’ confidentiality is assured.

Furthermore, the moderate sample size (N = 250) may have limited the statistical power and stability of the multivariable logistic regression models, particularly in estimating large odds ratios. This may have contributed to inflated effect estimates and wide confidence intervals for some predictors, such as WHR. Finally, the decision to exclude BMI from the regression models due to collinearity with BFM and WHR may have overlooked potential mediating or moderating effects among these variables. Future studies using mediation analysis or structural equation modeling could provide a more comprehensive understanding of the relationships between different anthropometric predictors of MetS.

### 4.2. Strengths

The strengths of the present study include the use of logistic regression analysis to evaluate the combined effect of various factors on the risk of MetS and its components. Focusing exclusively on the female population was also important, as this allowed us to consider gender-specific biological and social factors. Furthermore, the use of objective anthropometric measurements and clinical parameters, such as blood test results and blood pressure measurements, significantly increases the validity and reliability of the results obtained.

### 4.3. Future Research Directions and Practical Implications

It would be advisable to conduct longitudinal studies to assess the causal relationships between specific risk factors and the incidence of MetS. The analysis could also be expanded to include additional lifestyle variables, such as sleep quality and duration, stress levels, dietary habits, circadian rhythms, and detailed characteristics of smoking behaviour. Given the reliance on self-reported data for PA and smoking, future research would benefit from incorporating objective measurement methods (e.g., accelerometers, biochemical markers) to improve the validity and reliability of behavioural indicators. Another prospective research direction would be to evaluate the effectiveness of personalised preventive interventions aimed at modifying MetS risk factors in women. Future research should also aim to collect longitudinal data to assess the temporal and potentially causal relationships between sociodemographic, behavioural, and anthropometric factors and the development of MetS. Modeling trajectories of metabolic parameters over time could help to identify critical transition points and inform more effective prevention strategies. Furthermore, future research could also apply mediation or structural equation modeling techniques to explore the interrelationships among anthropometric indicators such as BMI, BFM, and WHR. This would allow for a deeper understanding of potential mediating or moderating effects in the development of MetS and address collinearity issues that limit the use of certain variables in traditional regression models. These results could inform the development of effective, targeted MetS prevention strategies with significant public health implications.

## 5. Conclusions

Metabolic syndrome (MetS) in women was strongly associated with age and parameters of visceral obesity, particularly the waist-to-hip ratio (WHR), which was found to be the most significant predictor of MetS. Body fat mass (BFM) also showed significant associations with most MetS components. Together, these two indicators emphasise the importance of accurately assessing body composition when diagnosing metabolic risk, rather than relying solely on BMI. Sociodemographic characteristics, such as place of residence, level of education, and occupational status, influence the risk of individual MetS components.

These results emphasise the significant role of obesity as a risk factor for MetS in women, highlighting the need for personalised preventive strategies that consider lifestyle and sociodemographic factors, such as targeted health education, promotion of physical activity, and dietary counselling adapted to the needs of women at risk.

## Figures and Tables

**Table 1:** Characteristics of the female study group.

Variable		Frequency	Percent (%)
Place of residence	Village	47	18.8
	City	203	81.2
Employment	Retirement or pension	63	25.2
	Non-working	1	0.4
	Casual work	12	4.8
	Permanent work	174	69.6
Education	Primary	1	0.4
	Lower secondary	4	1.6
	Vocational	9	3.6
	Secondary	65	26
	Higher	171	68.4
BMI	Underweight	4	1.6
	Normal body weight	79	31.6
	Overweight	81	32.4
	Obesity class I	55	22
	Obesity class II	21	8.4
	Obesity class III	10	4
Smoking	No	207	82.8
	Yes	43	17.2
Level of PA	Insufficient	111	44.4
	Sufficient	59	23.6
	High	80	32
TC	Normal	86	34.4
	Abnormal	164	65.6
HDL	Normal	197	78.8
	Abnormal	53	21.2
TG	Normal	158	63.2
	Abnormal	92	36.8
LDL	Normal	129	51.6
	Abnormal	121	48.4
Fasting glucose	Normal	135	54
	Abnormal	115	46
Non-HDL	Normal	96	38.4
	Abnormal	154	61.6
Blood pressure	Normal	131	52.4
	Abnormal	119	47.6
MetS	No	159	63.6
	Yes	91	36.4

BMI—body mass index; HDL—high-density lipoprotein; LDL—low-density lipoprotein; non-HDL— non–high-density lipoprotein cholesterol; TG—triglycerides; TC—total cholesterol; TG—triglycerides; PA—physical activity; MetS—metabolic syndrome.

**Table 2:** Values of individual parameters in the study group.

Variable	N	Min	Max	Me
Age	250	23	85	55
TC (mg/dL)	250	100	359	198
HDL (mg/dL)	250	15	109	58.5
TG (mg/dL)	250	45	385	120.5
LDL (mg/dL)	250	12	254	113
Fasting glucose (mg/dL)	250	69	158	98
non-HDL (mg/dL)	250	31	313	138.5
SBP (mmHg)	250	80	175	121.5
DBP (mmHg)	250	50	96	75
BMI (kg/m^2^)	250	16.7	47.6	27.2
Body weight (kg)	250	42.7	121.6	71.65
BFM (kg)	250	9.7	64.4	27.55
WHR	250	0.46	1.41	0.86

BMI—body mass index; BFM—body fat mass; DBP—diastolic blood pressure; FFM—fat free mass; HDL—high-density lipoprotein; LDL—low-density lipoprotein; Min—minimum; Max—maximum; Me—median; *N*—abundance; non-HDL—non–high-density lipoprotein cholesterol; SBP—systolic blood pressure; WHR—waist-to-hip ratio; TC—Total cholesterol; TG—triglycerides.

**Table 3:** Distribution of meeting MetS criteria according to body weight category.

MetS Components			BMI			Test Result
			Normal Body Weight	Overweight	Obesity	
Obesity criterion	No	*N*	58	25	1	*χ*^2^ = 96.177 *df* = 2 *p* = 0.001
		%	73.4%	30.9%	1.2%	
	Yes	*N*	21	56	85	
		%	26.6%	69.1%	98.8%	
Blood pressure criterion	No	*N*	52	48	29	*χ*^2^ = 19.266 *df* = 2 *p* = 0.001
		%	65.8%	59.3%	33.7%	
	Yes	*N*	27	33	57	
		%	34.2%	40.7%	66.3%	
Criterion fasting glucose	No	*N*	57	43	31	*χ*^2^ = 21.563 *df* = 2 *p* = 0.001
		%	72.2%	53.1%	36.0%	
	Yes	*N*	22	38	55	
		%	27.8%	46.9%	64%	
Non-HDL cholesterol criterion	No	*N*	30	30	34	*χ*^2^ = 0.113 *df* = 2 *p* = 0.945
		%	38.0%	37.0%	39.5%	
	Yes	*N*	49	51	52	
		%	62.0%	63.0%	60.5%	
MetS	No	*N*	71	56	29	*χ*^2^ = 57.664 *df* = 2 *p* = 0.001
		%	89.9%	69.1%	33.7%	
	Yes	*N*	8	25	57	
		%	10.1%	30.9%	66.3%	

*χ*^2^—test statistics; *df*—degrees of freedom; *N*—abundance; *p*—statistical significance.

**Table 4:** Results of logistic regression analysis for MetS and its components.

Variable	Level of the Variable	Odds Ratio (OR) for MetS and Its Components				
		MetS	Obesity	Elevated BP	Elevated Glucose	Elevated Non-HDL
Age	–	1.06 **	1.04	1.06 ***	1.05 ***	1.04 **
SB	–	1	1	1	1	1 *
PA	–	1	1	1	1	1
BFM	–	1.15	1.42 ***	1.06 **	1.06 **	1.02
WHR	–	356.97 **	5.89 × 10^30^ ***	0.27	1.09	5.59
Place of residence	Village	1	1	1	1	1
	City	2.53 *	0.05	2.65 *	0.90	1.74
Employment	Non-working	1	1	1	1	1
	Working	1.63	0.30	1.01	1.56	5.90 ***
Education	Secondary or less	1	1	1	1	1
	Higher	0.6	2.05	0.40 *	1.15	0.75
Smoking	No	1	1	1	1	1
	Yes	0.87	7.77	1.18	1.03	0.75

OR—odds ratio; MetS—metabolic syndrome; BP—blood pressure; BFM—body fat mass; WHR—waist-to-hip ratio; SB—sedentary behaviours; PA—physical activity. * *p* < 0.05; ** *p* < 0.01; *** *p* < 0.001. Note: The extremely high odds ratio for WHR may reflect statistical overfitting or separation and should be interpreted with caution.

**Table 5:** Estimated probability of MetS according to combinations of age, BFM and WHR.

MetS					
Age (Years)	BFM (kg)	WHR	*p*	*SE*	95% CI
42.55	18.5	0.73	0.02	0.01	0–0.07
42.55	18.5	0.85	0.04	0.02	0.01–0.11
42.55	18.5	0.97	0.07	0.04	0.02–0.2
42.55	27.84	0.73	0.06	0.03	0.02–0.18
42.55	27.84	0.85	0.12	0.05	0.05–0.26
42.55	27.84	0.97	0.22	0.08	0.10–0.42
42.55	37.18	0.73	0.19	0.09	0.07–0.44
42.55	37.18	0.85	0.33	0.11	0.16–0.56
42.55	37.18	0.97	0.51	0.12	0.29–0.72
55.74	18.5	0.73	0.04	0.02	0.01–0.10
55.74	18.5	0.85	0.07	0.03	0.03–0.16
55.74	18.5	0.97	0.14	0.05	0.06–0.28
55.74	27.84	0.73	0.12	0.05	0.05–0.25
55.74	27.84	0.85	0.22	0.06	0.13–0.34
55.74	27.84	0.97	0.37	0.08	0.23–0.52
55.74	37.18	0.73	0.33	0.1	0.16–0.55
55.74	37.18	0.85	0.51	0.08	0.35–0.66
55.74	37.18	0.97	0.68	0.07	0.52–0.81
68.93	18.5	0.73	0.07	0.03	0.03–0.18
68.93	18.5	0.85	0.13	0.05	0.06–0.27
68.93	18.5	0.97	0.24	0.08	0.12–0.44
68.93	27.84	0.73	0.21	0.07	0.1–0.4
68.93	27.84	0.85	0.36	0.07	0.23–0.51
68.93	27.84	0.97	0.54	0.08	0.38–0.7
68.93	37.18	0.73	0.50	0.12	0.29–0.71
68.93	37.18	0.85	0.68	0.08	0.51–0.81
68.93	37.18	0.97	0.81	0.06	0.67–0.9

Age, BFM, and WHR were analyzed at three levels: mean − 1 *SD*, mean, and mean + 1 *SD*. *p*—probability—predicted probability of MetS; *SE*—standard error; 95% CI—95% confidence interval; BFM—body fat mass; WHR—waist-to-hip ratio.

## Data Availability

The raw data supporting the conclusions of this article will be made available by the authors on request.

## Supplementary Materials

The following supporting information can be downloaded at: https://www.mdpi.com/article/10.3390/jcm14165911/s1, Table S1: Multicollinearity diagnostics for variables included in logistic regression models; Table S2: Estimated probability of fulfilling the obesity criterion depending on WHR and BFM; Table S3: Estimated probability of meeting the blood pressure criterion depending on place of residence, age, and BFM; Table S4: Estimated probability of meeting the elevated blood pressure criterion depending on education level, age, and BFM; Table S5: Estimated probability of meeting the elevated glucose criterion depending on age and BFM; Table S6: Estimated probability of meeting the elevated non-HDL cholesterol criterion depending on employment status, age, and sedentary behaviour (SB).
