# Supplementary material for: Predictors of Metabolic Syndrome in Polish Women—The Role of Body Composition and Sociodemographic Factors
Source: J Clin Med. 2025 Aug 21;14(16):5911. doi: 10.3390/jcm14165911 (PMC12387626; doi:10.3390/jcm14165911)
Supplement: Supplementary file 1 [file jcm-14-05911-s001.zip › jcm-3783116-supplementary.pdf]

Supplementary Table S1. Multicollinearity diagnostics for variables included in logistic regression models.

Explained variable	Explanatory variable	Collinearity statistics	
		VIF	Minimum tolerance
MetS	Age	1.98	0.51
	Sedentary behavior	1.18	0.85
	Physical activity	1.19	0.84
	BFM	1.51	0.66
	WHR	1.33	0.75
	Place of residence	1.11	0.90
	Employment	2.04	0.49
	Education	1.22	0.82
	Smoking	1.08	0.92
Obesity	Age	1.87	0.54
	Sedentary behavior	1.08	0.92
	Physical activity	1.25	0.80
	BFM	1.95	0.51
	WHR	2.43	0.41
	Place of residence	1.39	0.72
	Employment	1.72	0.58
	Education	1.30	0.77
	Smoking	1.33	0.75
Blood pressure	Age	1.57	0.64
	Sedentary behavior	1.11	0.90
	Physical activity	1.11	0.90
	BFM	1.33	0.75
	WHR	1.26	0.79
	Place of residence	1.14	0.87
	Employment	1.59	0.63
	Education	1.20	0.84
	Smoking	1.09	0.92
Glucose	Age	1.67	0.60
	Sedentary behavior	1.12	0.89
	Physical activity	1.15	0.87
	BFM	1.31	0.77
	WHR	1.23	0.81
	Place of residence	1.10	0.91
	Employment	1.77	0.56
	Education	1.21	0.83
	Smoking	1.09	0.92
non-HDL	Age	1.98	0.51
	Sedentary behavior	1.18	0.85
	Physical activity	1.19	0.84
	BFM	1.51	0.66
	WHR	1.33	0.75

Explained variable	Explanatory variable	Collinearity statistics	
		VIF	Minimum tolerance
	Place of residence	1.11	0.90
	Employment	2.04	0.49
	Education	1.22	0.82
	Smoking	1.08	0.92

VIF – variance inflation factor

The following supplementary tables present the estimated probabilities of meeting the diagnostic criteria for metabolic syndrome and its individual components, based on combinations of key predictors.

Supplementary Table S2. Estimated probability of fulfilling the obesity criterion depending on WHR and BFM

Obesity criterion				
**WHR**	**BFM (kg)**	***p***	***SE***	**95%CI**
0.73	18.5	0	0	0–0.02
0.73	27.84	0.01	0.01	0–0.13
0.73	37.18	0.16	0.19	0.01–0.74
0.85	18.5	0.64	0.24	0.19–0.93
0.85	27.84	0.98	0.02	0.84–1
0.85	37.18	1	0	0.98–1
0.97	18.5	1	0	0.99–1
0.97	27.84	1	0	1–1
0.97	37.18	1	0	1–1

WHR – waist-to-hip ratio; BFM – body fat mass; *p* – estimated probability; *SE* – standard error; CI – confidence interval;

WHR and BFM values are presented at three levels: mean –1 SD (low), mean (average), and mean +1 SD (high).

Supplementary Table S3. Estimated probability of meeting the blood pressure criterion depending on place of residence, age, and BFM.

Elevated blood pressure criterion					
**Place of residence**	**Age (years)**	**BFM (kg)**	***p***	***SE***	**95%CI**
Village	42.55	18.5	0.13	0.06	0.05–0.31
	42.55	27.84	0.21	0.08	0.09–0.41
	42.55	37.18	0.31	0.11	0.14–0.55
	55.74	18.5	0.24	0.08	0.12–0.44
	55.74	27.84	0.36	0.09	0.2–0.55
	55.74	37.18	0.49	0.11	0.29–0.69
	68.93	18.5	0.4	0.12	0.21–0.63
	68.93	27.84	0.53	0.1	0.33–0.72
	68.93	37.18	0.66	0.1	0.45–0.82
City	42.55	18.5	0.29	0.08	0.16–0.47

42.55	27.84	0.42	0.09	0.26–0.59
42.55	37.18	0.55	0.11	0.35–0.74
55.74	18.5	0.46	0.07	0.32–0.61
55.74	27.84	0.59	0.06	0.47–0.71
55.74	37.18	0.72	0.07	0.57–0.83
68.93	18.5	0.64	0.08	0.48–0.77
68.93	27.84	0.75	0.05	0.64–0.84
68.93	37.18	0.84	0.05	0.73–0.91

BFM – body fat mass; *p* – estimated probability; *SE* – standard error; CI – confidence interval;
Age and BFM values represent low (–1 SD), average (mean), and high (+1 SD) levels.

Supplementary Table S4. Estimated probability of meeting the elevated blood pressure criterion depending on education level, age, and BFM.

		**Elevated blood pressure criterion**			
**Education**	**Age (years)**	**BFM (kg)**	***p***	***SE***	**95%CI**
Secondary or lower	42.55	18.5	0.29	0.09	0.14–0.49
	42.55	27.84	0.41	0.1	0.23–0.61
	42.55	37.18	0.54	0.11	0.32–0.75
	55.74	18.5	0.45	0.09	0.29–0.63
	55.74	27.84	0.59	0.08	0.44–0.72
	55.74	37.18	0.71	0.08	0.54–0.83
	68.93	18.5	0.63	0.09	0.45–0.79
	68.93	27.84	0.75	0.06	0.6–0.85
	68.93	37.18	0.83	0.05	0.71–0.91
Higher education	42.55	18.5	0.14	0.06	0.06–0.29
	42.55	27.84	0.22	0.07	0.11–0.39
	42.55	37.18	0.32	0.10	0.16–0.54
	55.74	18.5	0.25	0.07	0.13–0.41
	55.74	27.84	0.36	0.07	0.23–0.52
	55.74	37.18	0.49	0.09	0.33–0.66
	68.93	18.5	0.41	0.1	0.23–0.61
	68.93	27.84	0.54	0.09	0.37–0.7
	68.93	37.18	0.67	0.08	0.49–0.81

p – estimated probability; *SE* – standard error; CI – confidence interval; BFM – body fat mass;
Age and BFM values correspond to low (–1 SD), average (mean), and high (+1 SD) levels.

Supplementary Table S5. Estimated probability of meeting the elevated glucose criterion depending on age and BFM.

Elevated glucose criterion				
**Age (years)**	**BFM (kg)**	***p***	***SE***	**95%CI**
42.55	18.5	0.18	0.06	0.09–0.33
42.55	27.84	0.28	0.07	0.16–0.45
42.55	37.18	0.42	0.1	0.24–0.61
55.74	18.5	0.31	0.07	0.19–0.45
55.74	27.84	0.44	0.06	0.33–0.57
55.74	37.18	0.59	0.07	0.44–0.72
68.93	18.5	0.47	0.09	0.31–0.64
68.93	27.84	0.61	0.07	0.48–0.73
68.93	37.18	0.74	0.06	0.61–0.84

BFM – body fat mass; *p* – estimated probability; *SE* – standard error; CI – confidence interval.
Age and BFM values correspond to low (–1 SD), average (mean), and high (+1 SD) levels.

Supplementary Table S6. Estimated probability of meeting the elevated non-HDL cholesterol criterion depending on employment status, age, and sedentary behaviour (SB).

Elevated non-HDL criterion					
**Employment**	**Age (years)**	**SB (min/day)**	***p***	***SE***	**95%CI**
Non-working	42.55	170.64	0.13	0.06	0.05–0.31
	42.55	348.96	0.18	0.08	0.07–0.38
	42.55	527.29	0.24	0.1	0.1–0.48
	55.74	170.64	0.21	0.07	0.1–0.38
	55.74	348.96	0.27	0.08	0.15–0.45
	55.74	527.29	0.36	0.1	0.2–0.56
	68.93	170.64	0.31	0.08	0.18–0.48
	68.93	348.96	0.4	0.08	0.26–0.56
	68.93	527.29	0.49	0.09	0.32–0.67
Working	42.55	170.64	0.46	0.08	0.32–0.62
	42.55	348.96	0.56	0.07	0.43–0.68
	42.55	527.29	0.65	0.07	0.51–0.77
	55.74	170.64	0.6	0.07	0.46–0.73
	55.74	348.96	0.69	0.05	0.58–0.79
	55.74	527.29	0.77	0.05	0.64–0.86
	68.93	170.64	0.73	0.08	0.56–0.85
	68.93	348.96	0.8	0.06	0.66–0.89
	68.93	527.29	0.85	0.05	0.72–0.93

BFM – body fat mass; SB – sedentary behaviour (minutes/day); p – estimated probability; SE – standard error; CI – confidence interval.
Age and SB values correspond to low (–1 SD), average (mean), and high (+1 SD) levels.

Supplementary Results – Brief Interpretation

The higher the BFM and WHR indices of the women studied, the higher the likelihood of meeting the obesity criterion (Table S2). Among rural residents aged 42.55 years with a BFM of 18.5 kg, the likelihood of meeting the blood pressure criterion was lowest ($p = 0.13$; $SE = 0.06$). Among urban residents aged 68.93 years with a BFM of 37.18 kg, the likelihood was highest ($p = 0.84$; $SE = 0.05$) (Table S3). The lower the education level and the older the respondents were, and the higher their BFM, the greater the likelihood of meeting the blood pressure criterion (Table S4). The older the respondents and the higher their BFM, the higher the likelihood of meeting the glucose criterion (Table S5). Among non-working women aged 42.55 years who engaged in 170.64 minutes of sedentary activity per day, the likelihood of meeting the non-HDL criterion was lowest at $p = 0.13$ and $SE = 0.06$. Among working women aged 68.93 years who engaged in 527.29 minutes of sedentary activity per day, the likelihood was highest at $p = 0.85$ and $SE = 0.05$. Having a job, being older, and engaging in more sedentary activity predisposed individuals to a higher risk of meeting the non-HDL criterion (Table S6).
